# The antimicrobial effect of *Rosmarinus officinalis* extracts on oral initial adhesion ex vivo

**DOI:** 10.1007/s00784-022-04400-5

**Published:** 2022-02-09

**Authors:** Mira Günther, Lamprini Karygianni, Aikaterini Argyropoulou, Annette Carola Anderson, Elmar Hellwig, Alexios Leandros Skaltsounis, Annette Wittmer, Kirstin Vach, Ali Al-Ahmad

**Affiliations:** 1 Department of Operative Dentistry and Periodontology, Medical Center, Faculty of Medicine, University of Freiburg, Hugstetter Str. 55, 79106 Freiburg, Germany; 2 Clinic of Conservative and Preventive Dentistry, Center of Dental Medicine, University of Zurich, Zurich, Switzerland; 3 Department of Pharmacognosy and Natural Products Chemistry, Faculty of Pharmacy, National and Kapodistrian University of Athens, Athens, Greece; 4 Institute of Medical Microbiology and Hygiene, Faculty of Medicine, University of Freiburg, Freiburg, Germany; 5 Institute for Medical Biometry and Statistics, Faculty of Medicine and Medical Center, University of Freiburg, Freiburg, Germany

**Keywords:** Initial biofilm, In situ, Alternative treatment, *Rosmarinus officinalis* extract, Antimicrobial effect, Live/dead staining, Culture technique

## Abstract

**Objective:**

In the last few decades, there has been a growing worldwide interest in the use of plant extracts for the prevention of oral diseases. The main focus of this interest lies in the identification and isolation of substances that limit the formation of microbial biofilm which plays a major role in the development of caries, periodontitis, and peri-implantitis. In this clinical ex vivo study, we investigated the antimicrobial effects of *Rosmarinus officinalis* extract against oral microorganisms within in situ initial oral biofilms.

**Materials and methods:**

Initial in situ biofilm samples (2 h) from six healthy volunteers were treated ex vivo with *R. officinalis* extract at concentrations of 20 mg/ml and 30 mg/ml. The number of viable bacterial cells was determined by counting the colony-forming units. All surviving bacteria were isolated in pure cultures and identified using MALDI-TOF and biochemical testing procedures. Additionally, live/dead staining in combination with epifluorescence microscopy was used for visualizing the antimicrobial effects in the initial biofilms.

**Results:**

The number of colony-forming units in the *R. officinalis*–treated biofilms was significantly lower than in the untreated controls (*p* < 0.001). The reduction range of log10 was 1.64–2.78 and 2.41–3.23 for aerobic and anaerobic bacteria, respectively. Regarding the bacterial composition, large intra- and interindividual variability were observed. Except for *Campylobacter* spp., the average amount of all bacterial taxa was lower after treatment with *R. officinalis* than in the untreated biofilms. A total of 49 different species were detected in the untreated biofilms, while only 11 bacterial species were detected in the *R. officinalis*–treated biofilms. Live/dead staining confirmed that the *R. officinalis*–treated biofilms had significantly lower numbers of surviving bacteria than the untreated biofilms.

**Conclusions:**

The treatment with *R. officinalis* extract has a significant potential to eliminate microbial oral initial biofilms.

**Clinical relevance:**

The results of this study encourage the use of *R. officinalis* extracts in biofilm control and thus in the treatment of caries and periodontitis as a herbal adjuvant to synthetic substances.

## Introduction

In dentistry, antimicrobial substances are used to prevent and treat bacteria-associated oral infections by reducing the oral biofilm [1]. New substances are increasingly sought due to growing primary and secondary resistances against conventional synthetically produced antimicrobial and antiseptic substances, e.g., chlorhexidine (CHX). Several studies show that, especially in the oral cavity, the prevalence of antibiotic-resistant bacterial strains is on the rise [2–5]. For this reason, the focus is shifting back towards plant-based alternatives, as plants and their extracts have been used as medicines for centuries. More than 300,000 plant extracts have been described to date [6, 7] and while many of these have a potential pharmacological application, their antimicrobial effects have only been studied in a few cases.

Biofilm formation, defined as an organizational form of oral microorganisms and salivary components that irreversibly attach to oral surfaces and are embedded in a matrix of extracellular polymeric substances (EPS), leads to a change in the properties of microorganisms compared to those in the planktonic form [8–11]. Thus, biofilms show significantly higher resistance to antimicrobial substances (100 to 1,000-fold) [2, 12, 13]. Oral biofilms are closely associated with diseases of the teeth and periodontium [14]. Although the genesis of inflammatory periodontal and carious diseases is multifactorial, the presence of an oral biofilm is a crucial etiological factor. Supragingival biofilms attached to tooth surfaces can lead to caries, whereas subgingival biofilms can lead to periodontal disease and peri-implantitis.

For the prevention and treatment of oral diseases, diverse antimicrobial substances are used that either lead to a reduction of oral biofilm or which selectively inhibit bacterial taxa associated with specific oral diseases. Substances that inhibit the growth and proliferation of microorganisms or prevent their secondary adhesion mechanisms such as coaggregation and coadhesion are also applied. Given the growing inefficacy of synthetically produced agents, plant-derived compounds have gained interest in the development of therapies aiming to control oral biofilms [15]. Many plants have an intricate defense system to protect them from fungal and bacterial attacks. Various antimicrobial metabolites such as phytoanticipins, which provide a chemical barrier against microbial attack, and phytoalexins, which are secondary metabolites with antimicrobial activity, can be produced as part of the plant defense system [16, 17]. Representatives of phytoalexins include flavonoids, glycosteroids, terpenoids, or polyphenols [6]. In addition, plants have the ability to produce endogenous antimicrobial peptides that have a broad spectrum of activity against microorganisms and a low level of resistance [6]. Natural substances such as coffee extracts [18, 19], tea extracts [20], cranberry extracts [21], cranberry juice concentrate [22], or Manuka honey [23] have been shown to reduce the total bacterial count of adherent microorganisms. They all exhibit anti-adhesive and antimicrobial activity based on different mechanisms. For example, a decrease in bacterial membrane hydrophobicity or reduced enzyme activity of fructosyl- and glycosyltransferase are proposed as explanations for the strong anti-adhesive properties and the pronounced inhibition of biofilm by cranberry extract [15]. Tea extracts, on the other hand, are thought to lead to lysis of the bacterial cell membrane, resulting in the death of the bacterium [24].

Rosemary (*Rosmarinus officinalis*) extracts have been studied as potential therapeutic agents against various diseases [25, 26]. Several studies showed that *R. officinalis* exhibits hepatoprotective, anti-hyperglycemic, antifungal, antitumor, and anti-ulcerogenic effects [27, 28]. In addition, *R. officinalis* is reported to have antioxidant and antimicrobial activities [27, 29]. These properties are thought to be due to the high content of phytochemicals such as carnosic acid, rosmarinic acid, or chlorogenic acid [27]. In organic rosemary extracts, diterpene derivatives, camphor, urolic acid, carnosic acid, rosmanol, and rosmarinic acid are considered the main phenolic compounds [28, 30]. The different phenolic compositions are responsible for the antimicrobial properties of rosemary extracts [27]. In the aqueous *R. officinalis* extract, carnosol, rosmanol, carnosic acid, methyl carnosate, and various flavonoids dominate [31].

Due to their high polyphenol content and its associated strong antioxidant effect, some extracts obtained from *R. officinalis* are already used in food preservation [28]. In previous studies, it was shown that a methanolic crude extract of *R. officinalis* can inhibit the growth of *Streptococcus mutans* [29]. In addition, the antimicrobial activity of aqueous and methanolic *R. officinalis* extracts against S*treptococcus sobrinus* and *Streptococcus sanguinis* has been reported. These bacterial species account for a large proportion of cariogenic biofilms. If targeted elimination or inactivation of these species was achieved, this could represent a fundamental step in the prevention of carious lesions.

Thus, it can be assumed that *R. officinalis* extract suppresses the growth of cariogenic streptococci, which would recommend its application for caries prevention [29]. To date, however, little is known about its use in dentistry [27]. It is unknown whether *R. officinalis* extract is effective against other oral bacterial taxa, for example, periodontal pathogens such as *Porphyromonas gingivalis* and *Prevotella intermedia.* Its potential as a natural antigingivitis or antiperiodontitis agent also needs further investigation.

Therefore, the aim of the present study was to investigate the effect of *R. officinalis* extract on the initial oral biofilm, which is the onset of biofilm formation in the oral cavity. We investigated the antimicrobial activity of *R. officinalis* extract and determined its influence on the survival and diversity of the bacterial species within an initial oral biofilm. For this purpose, initial oral biofilms were cultivated in situ on bovine enamel slabs and treated ex vivo with *R. officinalis* extract solution of different concentrations and control solutions. As a null hypothesis, it was assumed that the *R. officinalis* extract showed no significant antimicrobial and antibiofilm effect on initial oral biofilms.

## Materials and methods

### Extraction process and analysis (high-performance thin-layer chromatography and UPLC-HRMS and HRMS/MS)

The preparation of the extraction and the analysis were performed as previously described [32]. The investigated plant extract of *Rosmarinus officinalis L.* (Lamiaceae) was isolated from superficial plant parts collected from public ground in the Attica region of Greece (Lat.: 37°58′07.98″ N, Long.: 23°47′11.34″ E, elevation: 253 m). The University of Athens has permission to collect small amounts of plant parts for research purposes if they are not from endangered or protected species. A sample of the collected plant parts was deposited at the herbarium in the Department of Pharmacognosy and Natural Products Chemistry, Faculty of Pharmacy, National and Kapodistrias University of Athens under number: *R. officinalis*-KL 163. To isolate the extract, the collected plant parts were ground into a fine powder with homogeneous particle size (SCIS, Allenwest-Eac ltd) followed by a 15-min ultrasound-assisted extraction (Elma S 100H) with 100% methanol as the extraction solvent. Extraction took place for 15 min at room temperature and a ratio of 1:10 of plant per solvent. Finally, the extract was separated from the solvent by evaporation at 40 °C under reduced pressure (Buchi Rotavapor R-200). The prepared dry extract was provided by the Faculty of Pharmacy, National and Kapodistrias University of Athens, for the study of its antimicrobial activity against initial oral biofilms.

To analyze the composition of the extract, high-performance thin-layer chromatography (HPTLC) was performed at the Faculty of Pharmacy, National and Kapodistrias University of Athens, using a CAMAG® system. To prepare the sample, 10 mg of extract was added to 1 ml of methanol and this solution was subsequently applied to a 20 × 10 cm TLC plate (silica gel 60 F_254_, Merck) with the aid of an automatic sampler (ATS4, CAMAG). The process was controlled using VisionCats 2.3 software (CAMAG). The following settings were selected: application volume: 8 µl, number of lanes: 6, band length: 8 mm, distance from the lower edge: 8 mm, distance from the left and right edges: 20 mm, distance between the different tracks: 10.4 mm.

For fully automated development of the HPTLC plates, the ADC2 automated development chamber (CAMAG) was used under the following settings: chamber saturation: 20 min, preconditioning of the plate: 10 min at 33% relative humidity (MgCl_2_), drying of the plate: 5 min. The mobile phases used were dichloromethane, methanol, water (70:30:4; v/v/v) and ethyl acetate, methanol, formic acid, and water (50:10:7:1; v/v/v/v). Subsequent measurements and documentation were performed with the CAMAG® TLC Visualizer 2, taking images at 254 nm and 366 nm.

### Preparation of the extract

Powdered extract of *R. officinalis* (100 mg) was dissolved in 1000 µl dimethyl sulfoxide (DMSO) resulting in a concentration of 100 mg/ml. Complete dissolution of the extract was achieved by alternately heating in a water bath to 36 °C and vortexing for up to 20 min. This solution was used as a stock solution and was stored in a cool, dark place for a maximum of 48 h. On the day of the experiment, dilutions were made with phosphate-buffered saline (PBS) to obtain extract concentrations of 20 mg/ml and 30 mg/ml. A 0.2% CHX solution served as a negative control and a DMSO/PBS (1/10) solution and two 0.9% NaCl solutions as positive controls.

### Selection of the study participants

A total of six volunteers aged between 21 and 51 years were selected to participate in the study. All participants were in good general health. The following exclusion criteria were defined: (1) cardiovascular diseases, (2) blood coagulation disorders or blood diseases, (3) intake of medication, (4) allergy to the materials used for the splints, (5) pregnancy, (6) use of antibiotics or mouth rinses in the last 6 months prior to the start of the study, (7) suffering from xerostomia (dry mouth). The volunteers did not regularly consume alcohol or drugs and were nonsmokers. A healthy adult complete dentition or adult dentition with adequate prosthetic and/or conservative care was required to ensure adequate stability of the splint system and undisturbed bacterial attachment. All participants were free of active carious lesions or periodontal disease. The study participants were neither allowed to eat anything nor to perform oral hygiene measures 2 h prior and during initial biofilm formation. Before the start of the study, all participants gave their written informed consent. The study was approved by the Ethics Committee of the Albert-Ludwigs-University Freiburg (No. 502/13).

### Obtaining initial oral biofilm from intraoral splint systems

Bovine enamel slabs (BES) were used to generate an initial biofilm in vivo. For this purpose, bovine mandibular incisors were extracted from freshly slaughtered 2-year-old BSE (bovine spongiform encephalopathy)-free cattle at the Freiburg abattoir (Freiburg, Germany). Cylindrical enamel slabs (diameter of 5 mm) were punched from the labial surfaces of the teeth and reduced to a height of approximately 1.5 mm. The enamel side was ground to a flat surface using a wet disc grinder (Knuth-Rotor 3, Struers GmbH, Ballerup, Denmark) with 220 and 500 grit sandpaper and then finished and polished with 1200, 2400, and 4000 grit, as was described previously [33–35]. The specimens were ground so that no dentin was exposed on the surface. The BES had a thickness of around 1.0 mm and a diameter of 5 mm and thus an enamel surface area of 19.635 mm^2^. The BES were disinfected in 70% ethanol for at least 48 h.

As proof of sterility, the disinfected BES were placed in an NaCl solution (0.9%) in the ultrasonic bath for 3 min. The NaCl-treated biofilms were plated onto yeast-cysteine blood agar (HCB) plates and Columbia blood agar (CBA) plates, incubated accordingly and evaluated.

**Fig. 1:**
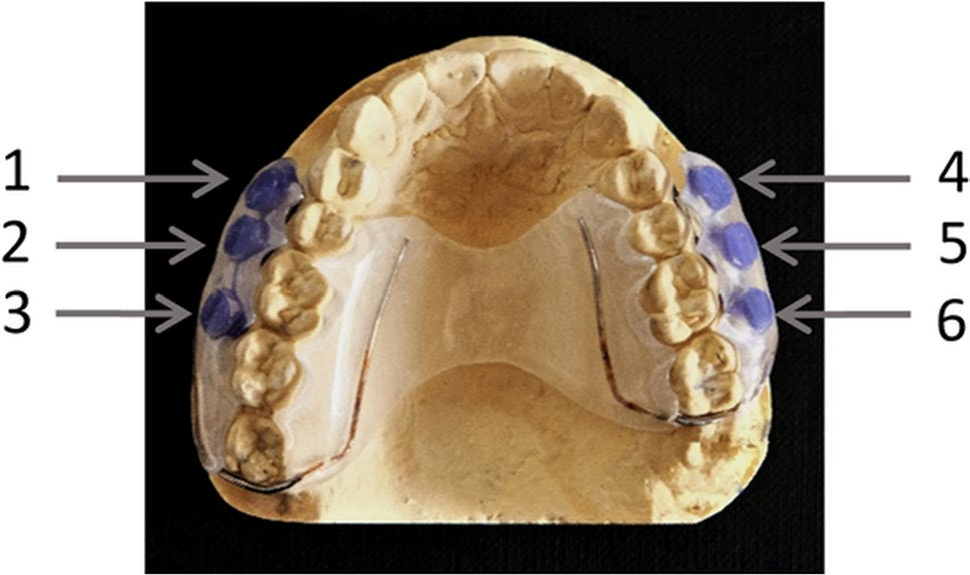
Individual upper jaw acrylic splint system. The BES (bovine enamel slabs) were attached at positions 1 to 6 of the splint with their upward-facing surfaces exposed to the buccal tooth surfaces. All other surfaces were covered with silicon

The BES were removed (26 h before further use), placed in a sterile Petri dish for 2 h to allow the ethanol to evaporate, and then placed in 0.9% NaCl solution for 24 h to hydrate [33, 35]. An individual intraoral acrylic splint system for the upper jaw was fabricated for each volunteer. It contained six retention wells on the inner surface of the vestibular acrylic components. The retentions were located in the approximal area between the first premolar and the second molar (Fig. 1). Before each experiment, the splint systems were disinfected with isopropyl alcohol and then rinsed with 0.9% NaCl solution. The BES were fixed in the wells using the addition-cured Silicone Panasil® (Kettenbach GmbH & Co. KG, Eschenburg, Germany) as previously described [33–36]. They were positioned so that only the enamel surface was in contact with the oral cavity. To avoid unwanted contact between the BES and the vestibular tooth surfaces, the gingiva, or the alveolar mucosa and to allow saliva flow, the vestibular area of the splint was bent 1 mm outward. All six volunteers carried the splint twice for 120 min.

### Protocol for the treatment of the initial biofilm with the R. officinalis extract

Each of the six volunteers wore the splint system with six BES twice for 120 min each in two different experiments. In total, 12 BES were treated with each concentration of *R. officinalis* extract. A total of 24 biofilm-covered BES were treated with 0.9% NaCl solution as a negative control, and 12 BES were treated with DMSO/PBS solution as a second negative control. After wearing, to ensure that all non-adherent bacteria were removed, the splints and BES were rinsed with 0.9% NaCl solution for 30 s. The BES were then removed from the splint system using sterile dental forceps without touching the surface covered with initial biofilm. One of the six biofilm-covered BES was placed in the sample extract of *R. officinalis* at a concentration of 20 mg/ml and one at a concentration of 30 mg/ml. One served as a positive control and was treated with 0.2% CHX. Two BES were treated with 0.9% NaCl and one with DMSO/PBS solution. These three served as negative controls. The BES covered with the initial oral biofilm were incubated for 10 min in the different treatment solutions consisting of *R. officinalis* extract as well as the controls. The test concentrations were determined in a pilot test, which showed that it is sufficient to evaluate the extract at concentrations of 20 mg/ml and 30 mg/ml, respectively. The BES were then rinsed again with 0.9% NaCl solution to clear them of extract or control solution residues. Subsequently, the BES were submitted to further microbiological and molecular biological test procedures.

### Quantification of the adherent oral microorganisms in the initial biofilms

The six treated BES were each added into Eppendorf tubes containing 1 ml 0.9% NaCl. They were treated for 3 min at 70% in an ultrasonic bath and subsequently vortexed for 60 s to enable the removal of the adherent microorganisms from the BES surfaces. A dilution series with 0.9% NaCl was then prepared for each sample up to a dilution of 1:10^3^ and 1:10^4^. The bacterial species were subsequently cultured and identified as previously described [37]. A 100-µl aliquot of each sample and dilution was plated onto one HCB plate and one CBA plate using a sterile glass spatula. The HCB plates served to cultivate and isolate anaerobic bacteria and were incubated in an anaerobic pot with a gas generator (GENbox, Biomerieux) to achieve an anaerobic environment at 37 °C for 7–10 days. The CoBl plates served to cultivate and isolate aerobic and facultative anaerobic bacteria and were incubated in an atmosphere with 5% CO_2_ for 3–5 days. Afterward, the number of colony-forming units (CFU) was determined. The quantification was performed using an optical system (WTW BZG 40, Weilheim, Germany).

### Identification of the adherent oral microorganisms within the initial biofilms

In order to differentiate the surviving microorganisms, the different colony types were determined based on their color and shape, odor, and hemolysis behavior, and their respective numbers were determined. Isolates were sub-cultivated to obtain pure cultures. For identification of these pure cultures, a MALDI-TOF analysis was performed in a MALDI BiotyperMicroflex LT (Maldi Biotyper, Bruker Daltonik GmbH, Bremen, Germany) as previously described [38]. The integrated Biotyper 3.0 software recorded mass spectra according to the manufacturer’s instructions. In addition, the software equates the recorded spectra with a reference database incorporating 3,740 reference spectra (319 genera and 1,946 species), whereby the results are listed as log score values. These values are used to indicate the validity of the results and correlate with the probability of correct species identification. Values of ≥ 2.0 were assumed to indicate species identification, and values ≥ 1.7 were assumed to indicate genus identification. For values below 1.7, no significant similarity with a spectrum deposited in the database could be detected. In cases where ambiguous results were obtained, the procedure was repeated.

### Live/dead staining and epifluorescence microscopy for visualization and quantification of initial biofilms

**Fig. 2:**
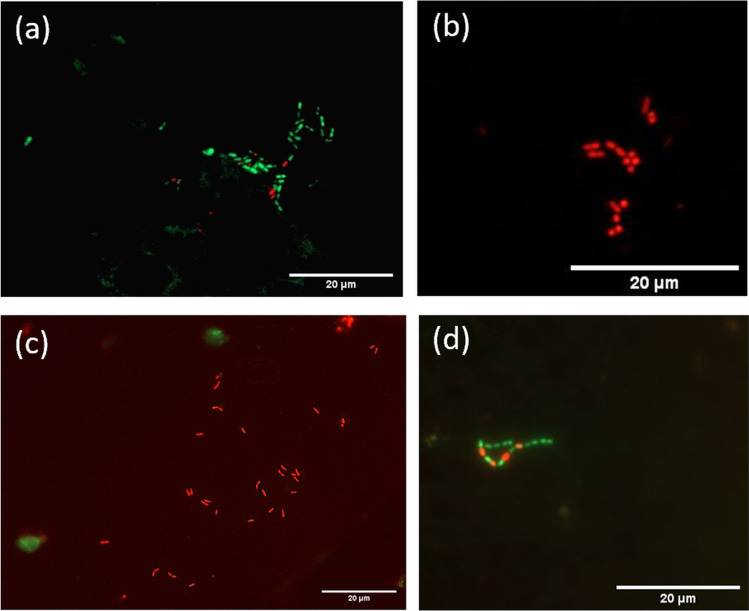
Epifluorescence microscopy images demonstrating the effect of *R. officinalis* on initial bacterial biofilm (2 h) examined by live/dead staining followed by fluorescence microscopy. Differentiation between live (green) and dead (red) microorganisms. Example images of **a** DMSO-treated biofilms, **b** *R. officinalis* (20 mg/ml)–treated biofilms, **c** CHX-treated biofilms, **d** NaCl-treated biofilms (negative control)

The fluorescent SYTO® 9 stain and propidium iodide (PI) assay (LIVE/DEAD® BacLight™ Bacterial Viability Kit, L7007, Life Technologies GmbH, Darmstadt, Germany) was applied to determine bacterial viability and to differentiate between live and dead bacteria within the biofilm samples as previously described [39]. Both stains, SYTO-9 and PI, were removed from the freezer (− 22 °C), thawed at room temperature, and mixed in a 1:1 ratio. Each BES was individually stained and microscopically examined. Staining solution (1 µl) was added to a well plate containing 500 µl NaCl, and the BES was placed inside and incubated for 10 min in a dark chamber at room temperature. After briefly washing it in 0.9% NaCl solution, it was placed on a special chamber slide (µ-Slide 8 Well, ibidi GmbH) that had a drop of 15 µl 0.9% NaCl on it. It was important to ensure that the dye-treated enamel slide was positioned so that the enamel side containing the initial biofilm was facing downward. Immediately thereafter, the Axio Observer.Z1 epifluorescence microscope (Carl Zeiss Microscopy, Jena, Germany) with a 63 × oil immersion objective was used for analysis. The Filter Set 38 HE was used to visualize the SYTO-9 staining (live bacteria), and the Filter Set 43 HE from Zeiss (Carl Zeiss Microscopy, Jena, Germany) was used for PI (dead bacteria). Images were recorded using the Zeiss AxioCam ICc5 CCD 5-megapixel camera (Carl Zeiss Microscopy, Jena, Germany). Ten images with 968 × 728 pixels were taken per sample. The scale per pixel was 0.144 µm × 0.144 µm, resulting in an image size of approximately 140 µm × 105 µm. The area of one image corresponds to 0.0147 mm^2^ of the sample surface. The individual images were taken using an ApoTome.2 system (Carl Zeiss Microscopy, Jena, Germany) which prevents stray light in the focal plane from being perceived as an interfering signal by detecting the magnification and pushing an appropriate grating into the beam path. Thus, stray fluorescence signals caused by unfocused light are reduced. Some exemplary images are shown in Fig. 2.

### Statistical analysis

The effects of treatment with *R. officinalis* extract were analyzed in a detailed evaluation for all six volunteers. In a descriptive analysis, the median, mean, and standard deviation were calculated. A graphical representation was performed using boxplots. Mixed linear models with random intercepts for each subject were fitted to compare the treatment groups. Here, because of the multiple measurements for each subject, the subject was considered a cluster. A correction was made for multiple testing using the Scheffe method. The significance level was set to *p* = 0.05. All statistical analyses were performed using the statistical software STATA 14.1.

## Results

### Effects of R. officinalis extract on the viable counts of oral microorganisms in initial biofilms

**Fig. 3:**
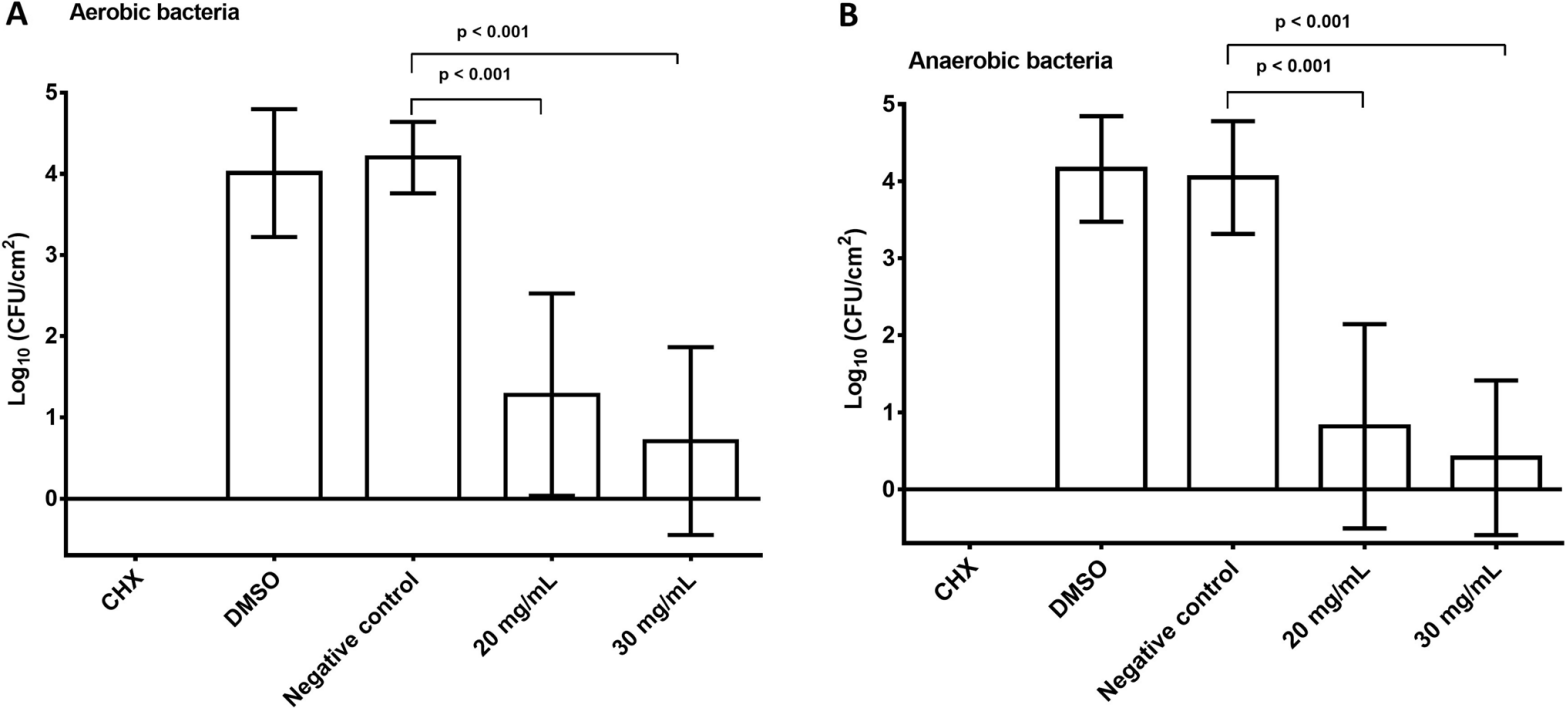
**A**, **B** Boxplots depicting colony forming unit (CFU) counts, showing the antimicrobial efficacy of *R. officinalis* against aerobic (**A**) and anaerobic (**B**) oral microorganisms after initial adhesion (2 h). The following treatment groups are shown: CHX-treated positive control, DMSO-treated neutral control, NaCl-treated negative control, *R. officinalis* extract at two different concentrations of 20 mg/ml and 30 mg/ml. The CFUs are presented on a log_10_ scale per square centimeter and the p-values are shown in the diagram

Figure 3 shows the efficacy of *R. officinalis* extract on the log counts of adherent oral microorganisms in the initial biofilms, as well as the untreated positive (CHX), negative (NaCl), and neutral (DMSO) controls. The *R. officinalis* extract displayed a pronounced antimicrobial effect on the total bacterial counts of initial microbial biofilms in situ. The untreated controls revealed mean log_10_ CFU/cm^2^ values of 4.20 ± 0.44 for initially adherent oral aerobic microorganisms and mean log_10_ CFU/cm^2^ values of 4.05 ± 0.73 for initially adherent oral anaerobic microorganisms; the neutral controls (DMSO) revealed mean log_10_ CFU/cm^2^ values of 4.10 ± 0.79 for aerobic microorganisms and 4.16 ± 0.68 for anaerobic microorganisms. The biofilms treated with CHX showed no bacterial growth, resulting in a 100% reduction. Incubation of initial biofilms with *R. officinalis* extract showed a significant reduction both in the concentration of 20 mg/ml with a mean decrease of 2.92 log steps to a mean value of 1.28 ± 1.24 log_10_ CFU/cm^2^ (*p* < 0.001 both) for aerobic bacteria and a mean decrease of 3.23 log levels to a mean value of 0.82 ± 1.32 log_10_ CFU/cm^2^ (*p* < 0.001 both) for anaerobic bacteria. At a concentration of 30 mg/ml *R. officinalis* extract, a mean decrease of 3.49 log levels to a mean value of 0.71 ± 1.16 log_10_ CFU/cm^2^ (*p* < 0.001 both) for aerobic bacteria and a mean decrease of 3.64 log levels to a mean value of 0.41 ± 1.01 (*p* < 0.001 both) for anaerobic bacteria could be detected.

### The shift of bacterial composition in the initial microbial biofilm after incubation with R. officinalis

**Fig. 4:**
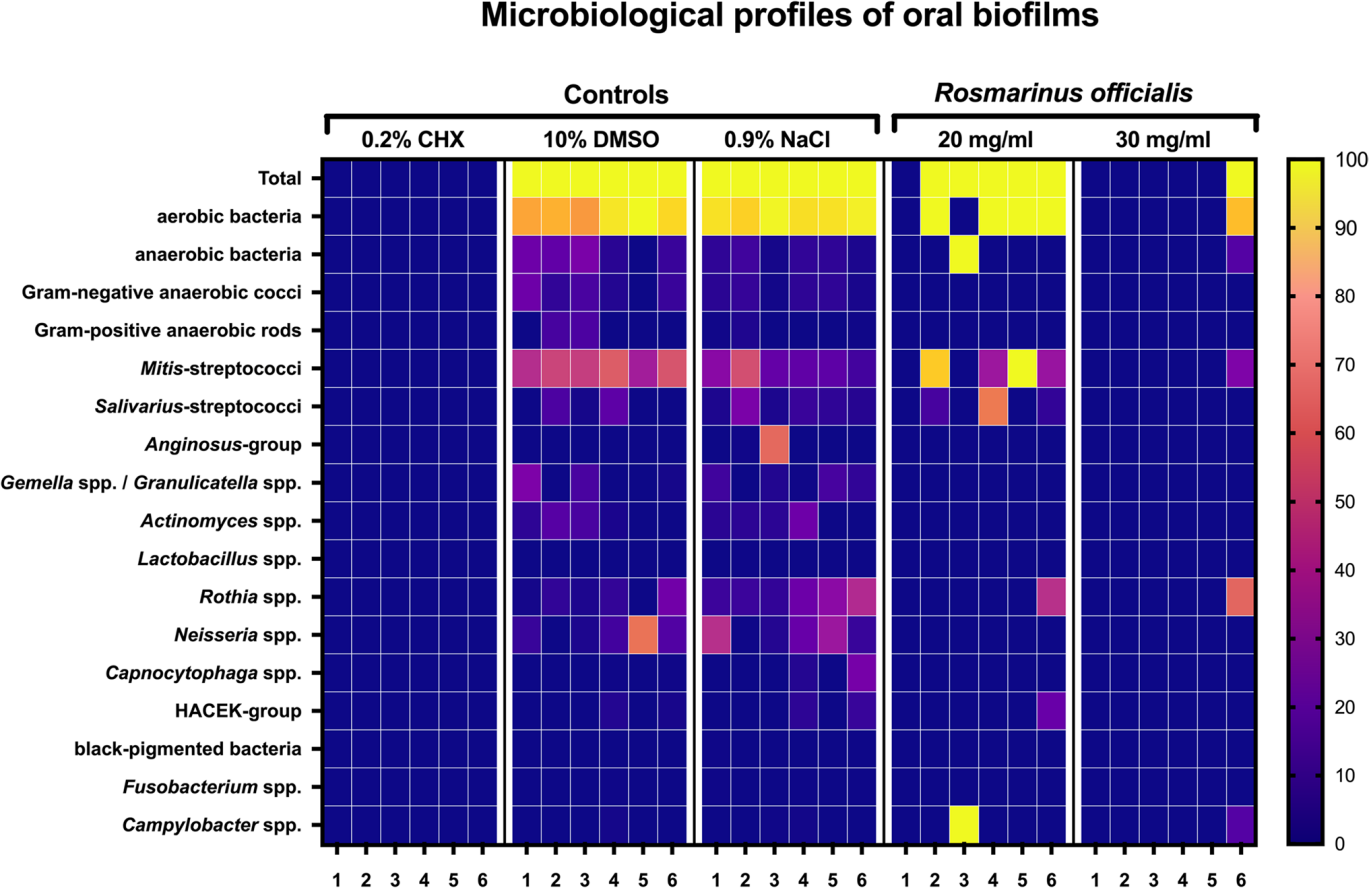
Heatmap demonstrating the absolute distribution (in log_10_/ml) of different bacterial groups/species among six study probands after a 2-h treatment with *R. officinalis* extract. An untreated negative control (NaCl 0.9%), a positive control (CHX 0.2%), and a control with DMSO (10%) were also used, as was the *R. officinalis* extract (20 mg/ml and 30 mg/ml) with a 10-min exposure time. The participant numbers for each treatment group are shown in columns and the variables (bacterial groups, species) in rows. The colors as depicted on the color scale bars on the right vary to indicate the change in data values for the different samples (low: 0–35, moderate: 35–70, high: 70–100)

**Fig. 5:**
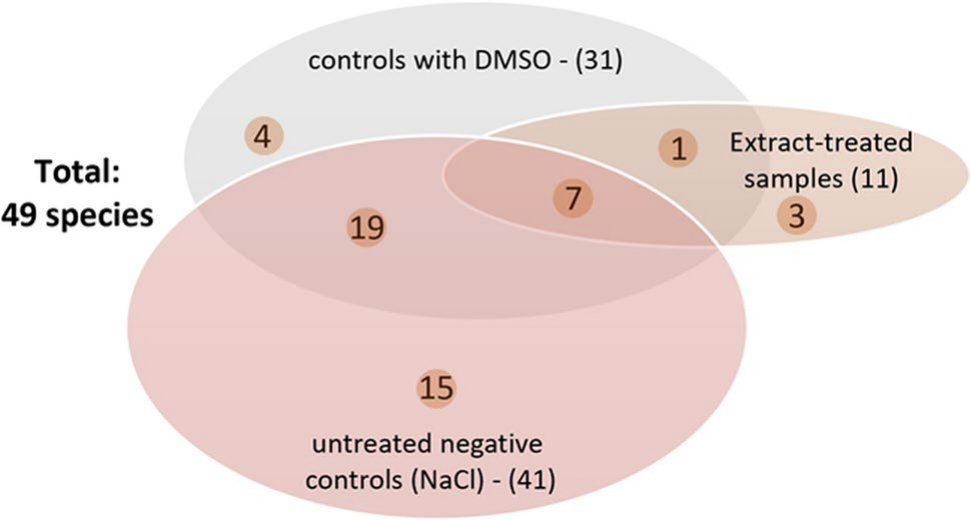
Diagram showing the distribution of bacterial species among six study probands. A total of 49 different species were detected: 41 different species in the untreated biofilms, 31 species in the DMSO controls, and only 11 different species in the *R. officinalis* extract-treated biofilms

**Fig. 6:**
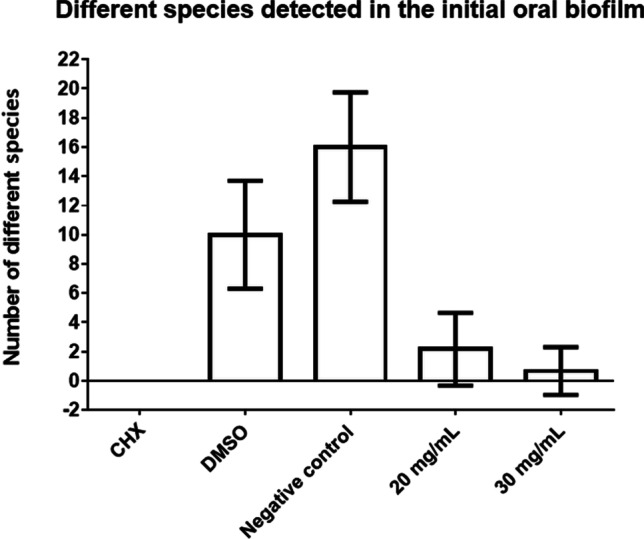
Average number of different bacterial species detected in initial oral biofilm treated with *R. officinalis* extract. The following treatment groups are shown: CHX-treated positive control, DMSO-treated neutral control, NaCl-treated negative control, *R. officinalis* extract at two different concentrations of 20 mg/ml and 30 mg/ml

The different bacterial species were determined for the cultivable bacteria of the untreated biofilms and were compared to the composition of the *R. officinalis* extract–treated samples. Figure 4 shows the composition of the initial biofilms of the untreated biofilms, the neutral controls (DMSO), and of the *R. officinalis* extract–treated biofilms (20 mg/ml and 30 mg/ml). In summary, a total of 49 different species could be detected in samples from six study participants with a clear dominance (30.92%) of 10 streptococcal species (*S. oralis*, *S. mitis*, *S. sanguinis*, *S. parasanguinis*, *S. gordonii*, *S. salivarius*, *S. vestibularis*, *S. anginosus*, *S. infantis*, *S. intermedius*). As shown in Fig. 5, in both untreated and neutral control biofilms, a total of 46 different bacterial species were identified, 38 of which were only isolated from the untreated biofilms. In the *R. officinalis* extract–treated samples, the number of different surviving bacterial species was reduced from 46 to 11 over six individuals. Three of these bacterial species could not be detected in the controls (NaCl or DMSO). The bacteria detected in the *R. officinalis* extract–treated biofilms included species of the genera *Streptococcus*, *Rothia*, *Haemophilus*, and *Campylobacter*. Thus, a significantly lower number of different species is present in the samples treated with *R. officinalis* extract (*p* < 0.05). Treatment with CHX killed all bacteria, thereby resulting in the elimination of the original bacterial community. Figure 6 shows the average amount of detected bacterial species among the six probands.

### Live/dead assay reveals the high bactericidal activity of R. officinalis extract against initial bacterial adhesion

**Fig. 7:**
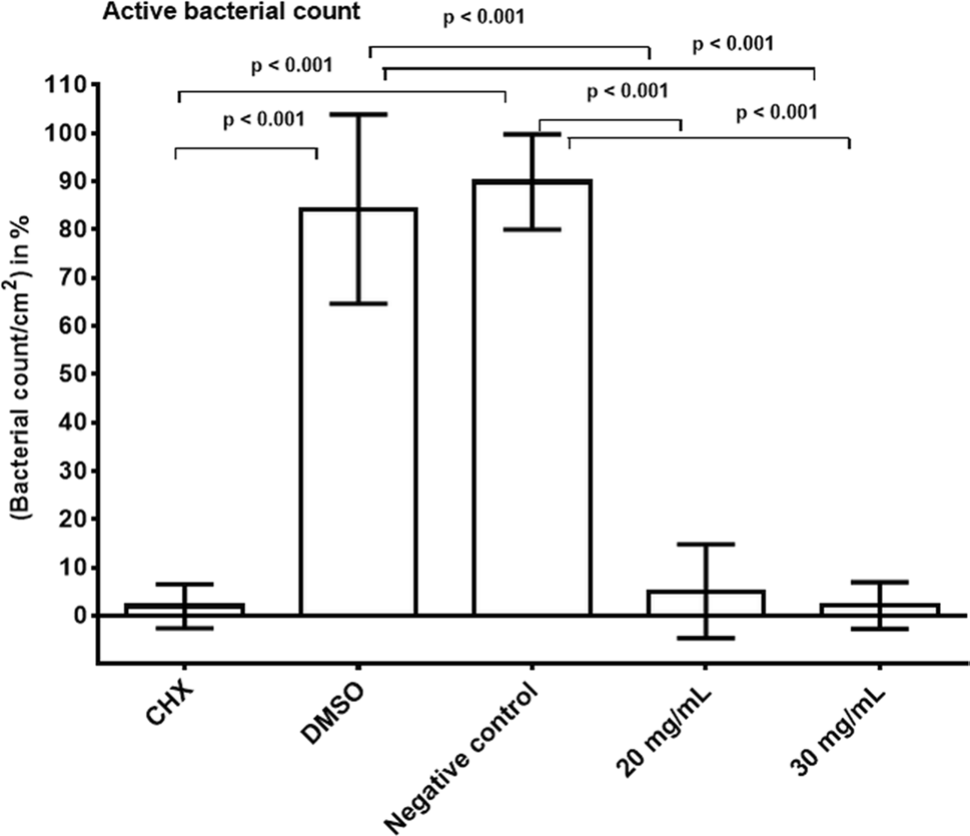
Boxplots showing the percentages of live bacteria on the BES surface after two-hour (2 h) oral exposure followed by treatment with *R. officinalis* extract and control solutions. The following treatment groups are shown: CHX-treated positive control, DMSO-treated neutral control, NaCl-treated negative control, *R. officinalis* extract at two different concentrations of 20 mg/ml and 30 mg/ml. The graph shows the bacterial counts per square centimeter as percentages and the *p*-values are shown in the diagram

The live/dead staining results are shown in Fig. 7. There was a mean percentage of 89.89% and 84.25% viable bacteria in the untreated initial biofilms of the negative (NaCl) and neutral controls (DMSO), respectively. The treatment of initial biofilms with *R. officinalis* extract significantly reduced these values to 5.05% at an extract concentration of 20 mg/ml and to 2.09% at an extract concentration of 30 mg/ml (*p* < 0.001). After treatment with 0.2% CHX, the vitality rate of viable bacteria in the initial biofilms was also significantly decreased to a mean percentage of 1.95% (*p* < 0.001). There was no significant difference between the positive control CHX and the extract-treated samples at either concentration (20 mg/ml and 30 mg/ml) (*p* = 0.758/*p* = 1.000).

## Discussion

Given the continuous global increase in antimicrobial resistance, the present study aimed to introduce a natural, plant-based antimicrobial agent for the prevention of oral diseases. For this purpose, the effect of *R. officinalis* extract on initial oral biofilms in situ was investigated. To the best of our knowledge, this study is the first to address the antimicrobial activity of *R. officinalis* extract against bacteria directly gained from the oral cavity in the form of in situ generated initial oral biofilm. Preventing the initial microbial adhesion is the most important step to avoid the severe consequences of mature biofilm formation, in which microorganisms are highly protected against the effects of different antimicrobials such as disinfectants and antibiotics [2, 13, 16, 40].

In the present study, a highly significant antimicrobial effect of *R. officinalis* extract was demonstrated on both aerobic and anaerobic bacteria of initial oral biofilms. The number of CFUs after initial biofilm treatment with *R. officinalis* extract was significantly lower than the CFUs of the untreated biofilms. Nevertheless, the CFUs were comparable to the CFUs yielded by the CHX-treated biofilms. Regarding the antibacterial properties of *R. officinalis* extract, other studies based on the minimal inhibitory concentration (MIC) and the minimum bactericidal concentration (MBC) also came to the same conclusion that *R. officinalis* extract has a bactericidal effect. For example, Sienkiewicz et al. [41] showed inhibition of *E. coli* growth with an *R. officinalis* essential oil. Various studies also confirmed the efficacy of *R. officinalis* against *S. aureus* [26, 42], *Bacillus cereus* [43], *Clostridium perfringens* [44], and various *Salmonella* species [45, 46]. In a study by Hickl et al. [32], the effect of several plant extracts including *R. officinalis* was tested on oral bacteria for the first time using in vitro models. The authors concluded that rosemary extract led to a significant reduction in the growth of all tested bacteria [32]. All of these studies used specific bacterial strains, whereas in the present study we chose an experimental setup that reflects the actual complexity and diversity of in situ oral biofilms in the oral cavity. By collecting in situ biofilms on enamel slabs, which are positioned intraorally on individual splint systems, the actual environmental parameters in the oral cavity (e.g., oxygen availability, pH, nutrient supply) can be integrated into the experimental model to better reflect the oral conditions and investigate the effect on a more diverse oral microbial spectrum of biofilms. This model has been used in previous studies and was proven to be sufficient for ex vivo studies [47–49]. Rosemary (*R. officinalis*) is a widely used plant, it is easy to grow, and inexpensive. In addition, studies have proven that *R. officinalis* extract is nontoxic and antimutagenic at the concentrations used [25, 50]. For example, the use of a 10-min mouth rinse with *R. officinalis* extract to minimize initial bacterial adhesion would be conceivable in dentistry. The fluorescence microscopy experiments after live/dead staining confirmed the microbiological results that *R. officinalis* extract has a significant disintegrating effect on initial biofilms. In principle, the finding that the number of green-stained live bacteria accounts for a high proportion in untreated initial biofilms is consistent with other studies [51, 52]. However, concerning the question of the percentages of live and dead bacteria present after live/dead staining, previous studies sometimes derived results that differed from those of the present work. For example, in a study by Tawakoli et al. [52], only 42 to 66% of vital bacteria were found in the initial biofilm after 120 min of intraoral exposure. The divergent results of the present work could be due to the fact that these are in situ studies in which individual differences are common due to a wide variety of factors. In addition, it could be due to the intraoral positions of the enamel slabs, which were positioned in different places in the various studies. Furthermore, it must be considered that fluorescence results after live/dead staining could depend on the temporary environment within the oral cavity of each volunteer.

The antimicrobial effect of *R. officinalis* extract was not only demonstrated by the reduction of the total bacterial counts and the reduced number of viable bacteria, but also by the fact that the total number of different detected bacterial species was significantly reduced after *R. officinalis* extract treatment. In the untreated biofilms, a total of 49 bacterial species out of 16 bacterial groups were detected. In the extract samples, there was a total of only 11 species out of five bacterial groups (*mitis* group streptococci, s*alivarius* group streptococci, *Rothia* spp., HACEK group, *Campylobacter* spp.). In all samples (excluding *R. officinalis* [30 mg/ml] and CHX-treated biofilms), non-mutans streptococci were the predominant taxa (DMSO: 52.44%, NaCl: 41.58%, *R. officinalis* 20 mg/ml: 56.41%), which is confirmed by previous studies [47, 53]. However, the absolute bacterial counts were significantly reduced by *R. officinalis* extract treatment. For a long time, mainly mutans streptococci were considered to be the relevant species in caries formation. However, recent studies assume that non-mutans streptococci may also have a significant impact on caries initiation [47, 54], as the increase in acidogenic non-mutans streptococci is considered to precede the attachment of mutans streptococci and other cariogenic germs. According to this hypothesis, reducing non-mutans streptococci in the initial biofilm could help prevent the development of carious lesions.

The presence of *Actinomyces* spp. was reduced by 100% by the *R. officinalis* extract treatment. Similar to non-mutans streptococci, *Actinomyces* spp. are able to produce acids that increase the risk of demineralization of dental enamel [47, 54]. In particular, large amounts of non-mutans streptococci and *Actinomyces* spp. have been detected in white spot lesions [55]. Again, the present results indicate that *R. officinalis* extract could be used for caries prevention by reducing the concentration of *Actinomyces* spp. in the oral biofilm. Interestingly, mutans streptococci were not detected in any of the study participants’ biofilms. Due to various virulence factors, *S. mutans* is considered to play a particular role in the development of caries [56]. The fact that no mutans streptococci were detected in the present study may be attributed to the fact that the microbial composition was examined after only 2 h of initial microbial colonization. Previous studies also showed that the proportion of mutans streptococci in initial biofilms was very low, at 2% or even less [53].

All results showed large intraindividual and interindividual differences, which is in line with previous studies investigating initial oral biofilms [20, 33, 51]. The composition of a biofilm is highly dependent on local or individual factors [57] such as diet, salivary flow rate, and salivary pH. These factors may differ from subject to subject, and could also vary within the oral cavity, for example, depending on the position of the enamel slabs. Therefore, it was ensured that the enamel slabs were always placed in the same position. Here, the *R. officinalis* extract samples were opposite the control samples in the splint system.

The present study has some limitations which should be considered in future investigations of this topic. One limitation of the present study is that only one treatment time over 10 min was tested. This long treatment time was intended to simulate the intensive use of *R. officinalis* ingredients in tea preparation. However, the inclusion of shorter rinse times such as 1 min in future studies would allow for a comparison with other mouth rinses, which are usually only used for 1 min in the oral cavity. Another limitation is the small number of probands involved in the study, as the number of microbial species isolated using the culture technique would have been increased if biofilm samples from a larger number of volunteers would have been studied. In addition to its activity against initial oral biofilm, the effects of *R. officinalis* extract against the oral biofilm formed over longer periods is an important aspect that should also be included in follow up-studies. Furthermore, testing the minimal inhibitory concentrations of the extract against a diverse array of oral bacterial species would complement the data of the present study in addition to the aspects highlighted here.

Further clinical studies on biofilms grown over a longer period are needed to investigate whether there is also a comparable antimicrobial effect of *R. officinalis* extract in such cases. In this study, the investigations were performed on 2-h-old initial biofilms. With the aid of a similar experimental set-up, in particular, through the use of intraoral splint systems, a mature biofilm of 2–3 weeks could be obtained for further investigations with *R. officinalis* extract. A determination of whether there is efficacy against other oral species, e.g., periodontal pathogens such as *Porphyromonas gingivalis* and *Prevotella intermedia*, and whether *R. officinalis* extract can be used as a natural substance in the prevention of periodontitis, requires further investigation in clinical studies including appropriate patient collectives.

## Conclusion

In conclusion, the antimicrobial efficacy of *R. officinalis* extract may be applied in preventive dentistry in the future. As shown in this study, the investigated *R. officinalis* extract has demonstrated a highly significant antimicrobial effect on bacteria of the initial oral biofilm and thus has a high potential for the prevention and treatment of oral diseases such as caries and periodontitis. The potential of using mouthwashes including *R. officinalis* extract for the prevention and treatment of caries, periodontitis, and peri-implantitis should be evaluated in further clinical studies.
